# Liquid biopsy proteomics of uveal melanoma reveals biomarkers associated with metastatic risk

**DOI:** 10.1186/s12943-021-01336-4

**Published:** 2021-02-24

**Authors:** Gabriel Velez, Huy V. Nguyen, Teja Chemudupati, Cassie A. Ludwig, Marcus Toral, Sunil Reddy, Prithvi Mruthyunjaya, Vinit B. Mahajan

**Affiliations:** 1 Molecular Surgery Laboratory, Stanford University, Palo Alto, CA USA; 2 Department of Ophthalmology, Byers Eye Institute, Stanford University, Palo Alto, CA 94304 USA; 3 Medical Scientist Training Program, University of Iowa, Iowa City, IA USA; 4 Division of Oncology, Department of Medicine, Stanford University, Palo Alto, CA USA; 5 Veterans Affairs Palo Alto Health Care System, Palo Alto, CA USA

**Keywords:** Proteomics, Vitreous, Liquid biopsy, Uveal melanoma, Cancer, Biomarker, SCFR, HGFR, STAT3

## Main text

Uveal melanoma (UM) is the most common primary intraocular tumor in adults [1]. Approximately 50% of patients with UM develop metastatic disease and up to 85% of these patients succumb to their visceral metastases. Currently, patients are screened with serial body imaging, which typically presents 2–4 years after primary diagnosis. However, it is suspected that micro-metastatic disease may develop 1–5 years prior to detection. Determining which patients are likely to metastasize has been the focus of recent molecular testing. To determine patients at higher risk for developing metastatic disease, clinical or histopathologic risk factors are considered [2]. More recently, gene expression analysis (i.e., gene expression profiling [GEP] and preferentially expressed antigen in melanoma [PRAME] status) after direct surgical tumor sampling has enhanced prognostic accuracy [3–5]. While tumor biopsies are beneficial for assessing mortality risk, they are still subject to certain risks (e.g., risk of retinal detachment and tumor dissemination) and not amenable to repeat testing [3–5]. Furthermore, they do not identify protein targets to direct new adjuvant treatments. In contrast, liquid biopsies offer a minimally invasive method for real-time molecular assessment of the primary tumor. Diagnostic vitrectomies are reproducible, repeatable, and carry a lower risk of adverse outcomes since they do not require invasion of the primary tumor. Serial fluid biopsies from the eye might allow prospective metastatic surveillance.

**Fig. 1:**
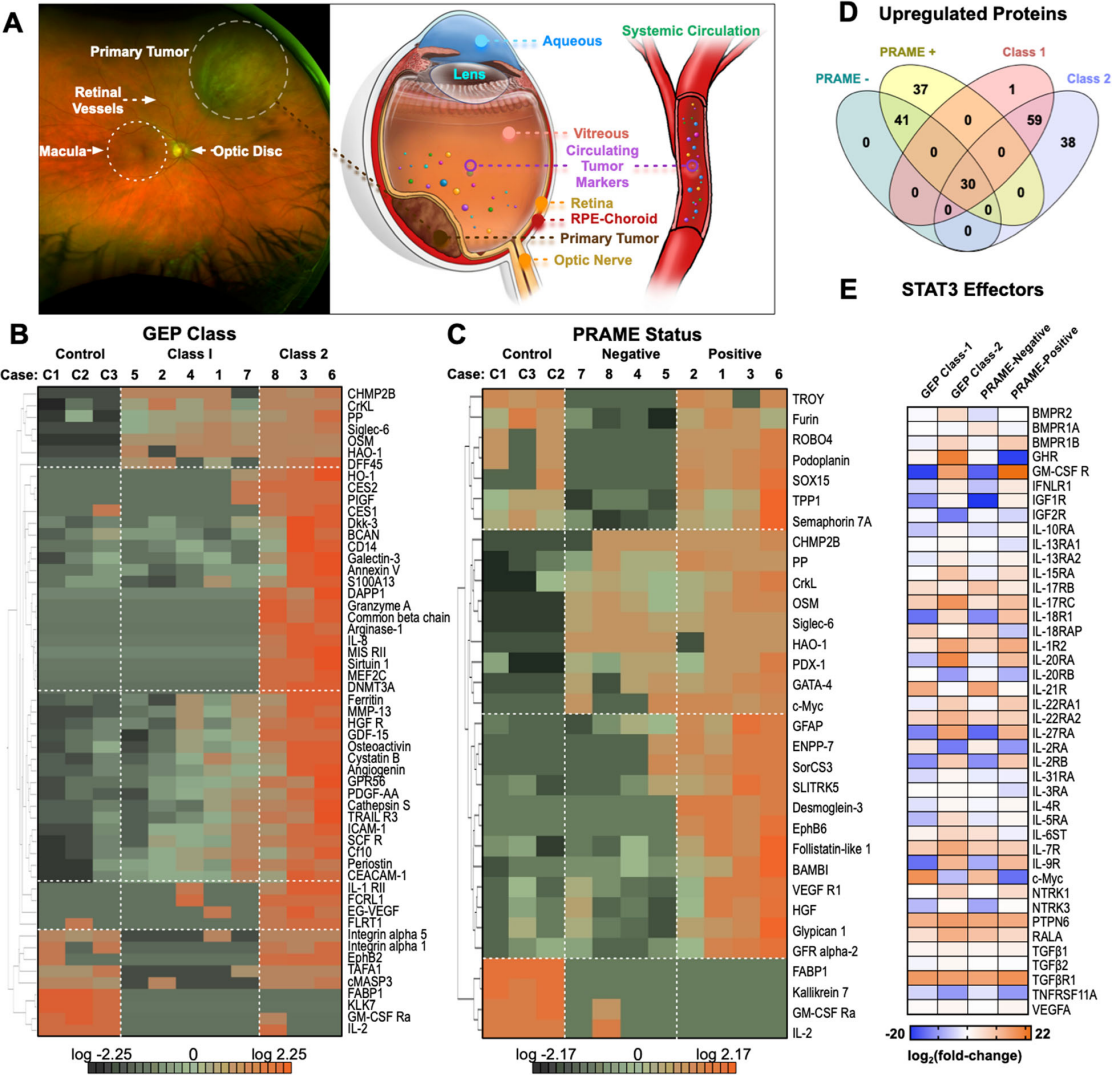
Targeted proteomic signatures differentiate molecular classes of uveal melanoma: **a** The vitreous contains native vitreous proteins, systemic protein biomarkers, and tumor biomarkers that can be sampled through proteomic analysis. Primary tumors can release circulating tumor markers into the vitreous and systemic circulation. Graphical illustrations by Alton Szeto and Vinit Mahajan. Permission to publish granted by original artist. **b** Protein concentrations from the multiplex ELISA array were normalized to log base 2 and analyzed for differentially expressed proteins. Multi-group comparison (1-way ANOVA) followed by hierarchical heat map clustering was used to identify differentially expressed proteins in the large-scale dataset. When comparing protein expression by GEP class, there were 46 differentially expressed proteins at the *p* < 0.01 level. Results are represented as a heatmap and display protein expression levels on a logarithmic scale. Orange indicates high expression while dark green/black indicates low or no expression. **c** When comparing protein expression by PRAME status, there were 32 differentially expressed proteins at the *p* < 0.01 level. **d** Comparative analysis of significantly upregulated proteins in each group (compared to controls) using Venn diagrams. **e** Expression of STAT3 signaling effectors identified in UM vitreous. Results are represented as a heatmap and display protein expression levels on a logarithmic scale. Samples are grouped according to their respective GEP class or PRAME status. Orange indicates high expression while dark blue indicates low or no expression

Global protein changes may provide an independent biomarker that reflects the total sum of the tumor effects or a supplemental biomarker that could enhance diagnostic sensitivity and specificity. Our research has demonstrated that retinal proteins leak into the vitreous, and that proteomic analysis of vitreous biopsies can detect molecular changes in the adjacent retina, uncover biomarkers, and identify protein targets for adjuvant therapy [6]. These biomarkers are concentrated in fluid compartments adjacent to the primary tumor (Fig. 1a). There have been proteomic studies on UM tumor cells which have suggested differentially expressed proteins (DEPs), but liquid protein markers have yet to be validated (Table S1). Few studies utilized quantitative platforms and none, to date, measure protein biomarkers in the vitreous. In this study, we use proteomics to detect candidate biomarkers for UM and identified targets for drug repositioning.

### Targeted proteomic analysis distinguishes molecular classes of UM

A total of eight patients were diagnosed with UM and used for the discovery phase analysis. The clinical and demographic information are described in Table S2 [4, 7, 8]. Vitreous biopsies were collected in the operating room (see Methods). For patients undergoing I-125 plaque brachytherapy, vitreous biopsies were collected prior to placement of the active plaque and before tumor biopsy. No biopsy complications were observed. In enucleated eyes, vitreous collection was performed following trans-scleral tumor biopsy (without vitreous violation) prior to formalin fixation. There were 77 proteins DEPs among control and UM vitreous (62 upregulated and 15 downregulated proteins; *p* < 0.05). Among the proteins upregulated in UM vitreous were SIGL6, c-Myc, and SCFR/c-Kit. FABP1 and KLK7 were highly expressed in control vitreous. When comparing protein expression by GEP class, there were 46 DEPs (*p* < 0.01; Fig. 1b; Table S3). Among the upregulated proteins in GEP Class-2 patients were HGFR/c-MET, SIRT1, DNMT3A, common β-chain (βc), ARGI1, and FasL. When comparing by PRAME status, there were 32 DEPs (*p* < 0.01; Fig. 1c-d; Table S4). Among the upregulated proteins in PRAME-positive patients were DSG3, ENPP-2, LEG9, and HGF. The top-represented pathway detected in UM vitreous was the STAT3 pathway (*p*-value = 0.004; activation z-score = 2.7; Fig. 1e). Forty-two downstream STAT3 signaling effectors were differentially expressed. Recent transcriptomic analysis of UM liver metastases detected *STAT3* upregulation [9]. Interestingly, vitreous from patients with GEP Class-2 and PRAME-positive tumors displayed higher representation of STAT3 signaling proteins. Taken together, these results identified potential vitreous proteins that correlate with gene expression patterns for UM metastatic risk.

### Biomarker validation study

**Fig. 2:**
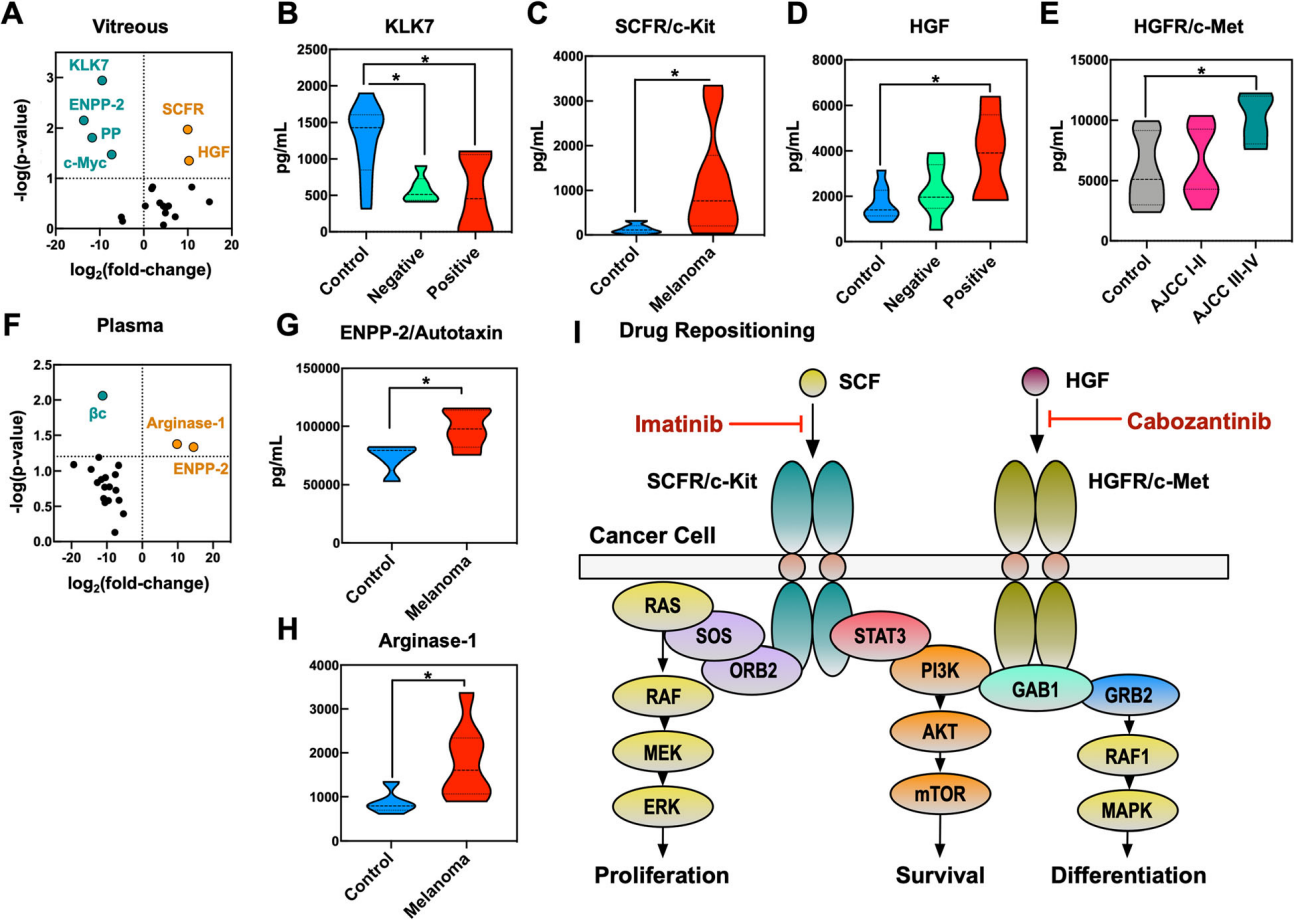
Biomarker verification study confirms expression of SCFR, HGF, and HGFR in UM vitreous: Prospective verification of 20 biomarkers in paired vitreous (UM, *n* = 11; control, *n* = 11) and plasma (UM, *n* = 8; control, *n* = 5). **a** Vitreous differential protein expression data are represented as a volcano plot. The horizontal axis (x-axis) displays the log2 (fold-change) in protein expression (UM vs. controls) and the vertical axis (y-axis) displays the noise-adjusted signal as the -log10 (*p*-value) from by Student’s pairwise t-test (Alpha = 0.05). **b** Expression of KLK7 sorted by PRAME status (i.e., PRAME-positive or -negative), **c** SCFR/c-Kit, **d** HGF sorted by PRAME status and **e** HGFR/c-Met in patient vitreous. **f** Plasma differential protein expression data are represented as a volcano plot. Expression of **g** ENPP-2 and **h** ARGI1 in patient plasma. Expression data is represented as protein concentration (pg/mL) and displayed as mean ± SEM. Data were analyzed by 1-way ANOVA followed by Tukey’s multiple comparison test or by Student’s pairwise t-test, where appropriate (Alpha = 0.05). **i** Schematic representation of verified UM vitreous biomarkers (SCFR/c-Kit, HGF, and HGFR/c-Met) and their role in pathways mediating cancer cell proliferation, survival, and differentiation. Imatinib (SCFR inhibitor) and cabozantinib (HGFR inhibitor) could be repurposed for adjuvant UM therapy

To prospectively validate the discovery dataset, we examined an independent cohort of patients. Twenty proteins were selected for further study based on their statistical significance and biological function (Table S5). We generated a custom ELISA that measured these 20 proteins in control (*n* = 11) and UM (*n* = 11) vitreous, as well as in control (*n* = 5) and UM plasma (*n* = 8; Fig. 2a). The clinical and demographic information are described in Table S6. KLK7, a negative control marker, was decreased in UM vitreous compared to controls, as predicted (*p* = 0.001; Fig. 2b). We also confirmed the presence of elevated SCFR (*p* = 0.011) and HGF (*p* = 0.044) in UM vitreous (Fig. 2c-d). When analyzed by genetic class, we observed an increase in c-Myc levels in GEP Class-2 vitreous compared to GEP Class-1A (*p* = 0.009; Figs. S1, S2 and S3). We observed elevated HGFR in AJCC Stage-III and -IV UM vitreous (*p* = 0.046; Fig. 2e). Interestingly, ENPP-2 levels were increased in UM vitreous in the training dataset but decreased in the validation cohort compared to controls (*p* = 0.02; Table S7). These results support the verification of HGF, HGFR, and SCFR expression in UM vitreous. ENPP-2 and ARGI1 levels were significantly elevated in UM plasma (*p* = 0.044 and 0.042, respectively; Fig. 2f-h; Table S8). Despite levels of βc being elevated in UM vitreous in the training dataset, βc levels were decreased in the UM validation cohort (Fig. 2f). These results suggest that ENPP-2 and ARGI1 could serve as independent plasma biomarkers for UM. Prospective, serial studies are needed to further validate their expression in the UM plasma proteome.

## Discussion

Advances in molecular genetic testing have enhanced the prognostic accuracy and management of UM [7, 9]. The current prognostic classification of UM is performed by direct gene expression measurements in primary tumor surgical tissue. However, these tumor biopsies are invasive and not yet validated for repetitive serial testing and follow-up [10]. We therefore sought to identify vitreous proteins that could serve as accessible biomarkers in place of tumor genes. We found that vitreous biopsies may provide additional or improved diagnostic information without disrupting the tumor. UM vitreous displayed elevated expression of HGFR, HGF, and SCFR and lower expression of KLK7. HGFR has previously been reported to be upregulated in UM tumor cells [11]. Future studies could investigate patient-specific trends using serial liquid biopsies. We cross-referenced databases to identify DEPs targeted by FDA-approved drugs (Fig. 2i). SCFR is inhibited by imatinib. HGFR/c-MET signaling is inhibited by cabozantinib, which was reported to improve median overall survival in a phase-II randomized discontinuation trial for metastatic melanomas [12]. Our results suggest that it may be valuable as an earlier intervention. SCFR and HGFR may thus become drug repositioning candidates for adjuvant UM therapy.

Limitations to the current study including limited sample number, high number of measurements, and intra-tumor heterogeneity. However, our prospective analysis aided in verifying several statistically significant vitreous biomarkers in a separate cohort. The large proportion of albumin, which may dilute biomarker concentrations, and the wide range of abundance of other proteins can make plasma difficult to characterize. Proteomic changes in plasma are dynamic and may represent a variety of unrelated systemic changes. Thus, further verification in a larger cohort is required.

## Conclusions

Vitreous biopsies can be used to identify candidate biomarkers for UM. Moreover, proteomic profiles can suggest biologically plausible mechanisms for tumor proliferation and suggest rational approaches for adjuvant therapy and metastatic risk surveillance. These biomarkers could help rationally design future clinical trials, including for previously untreatable micro-metastatic UM, and may provide prognostic information comparable to primary tumor gene expression.

## Supplementary Information

**Additional file 1.**

## Data Availability

Further information and requests for resources and reagents should be directed to and will be fulfilled by the Lead Contact, Vinit B. Mahajan (vinit.mahajan@stanford.edu). All unique/stable reagents generated in this study are available from the Lead Contact with a completed Materials Transfer Agreement.

## Abbreviations

AJCC: American Joint Committee on Cancer
ARGI1: Arginase-1
βc: Common β-chain
DEP: Differentially expressed protein
DNMT3A: DNA (cytosine-5)-methyltransferase 3A
DSG3: Desmoglein-3
ELISA: Enzyme-linked immunosorbent assay
ENPP-2: Ectonucleotide pyrophosphatase/phosphodiesterase 2
FABP1: Fatty acid-binding protein 1
FasL: Fas ligand
GEP: Gene expression profile
HGF: Hepatocyte growth factor
HGFR: Hepatocyte growth factor receptor
KLK7: Kallikrein-7
LEG9: Galectin-9
PRAME: Preferentially expressed antigen in melanoma
SCFR: Stem cell factor receptor
SIGL6: Siglec-6
SIRT1: Sirtuin-1
STAT3: Signal transducer and activator of transcription 3
UM: Uveal melanoma
