# Supplementary material for: Liquid biopsy proteomics of uveal melanoma reveals biomarkers associated with metastatic risk
Source: Mol Cancer. 2021 Feb 24;20:39. doi: 10.1186/s12943-021-01336-4 (PMC7903662; doi:10.1186/s12943-021-01336-4)

Supplementary Online Content

Liquid biopsy proteomics of uveal melanoma reveals biomarkers associated with metastatic risk

Table of Contents

Methods

Case Series

Supplemental References

Table S1. Proteomic studies on uveal melanoma using human tissue

Table S2. Patient demographics for training dataset

Table S3. Differentially expressed proteins based on GEP class

Table S4. Differentially expressed proteins based on PRAME status

Table S5. Vitreous biomarkers for verification

Table S6. Patient demographics for verification study

Table S7. Vitreous biomarker verification

Table S8. Plasma biomarker verification

Fig. S1. Vitreous biomarker levels in training dataset

Fig. S2. Vitreous biomarker levels in verification dataset arranged by GEP Class

Fig. S3. Vitreous biomarker levels in verification dataset arranged by PRAME Status

Fig. S4. Vitreous biomarker levels in verification dataset arranged by AJCC Stage

Methods

The prospective case control study was approved by the Stanford University Institutional Review Board (IRB)/Ethics Committee and adhered to the tenets set forth in the Declaration of Helsinki. All participants provided written informed consent. Data were collected and analyzed from November 2018 to March 2020.

Clinical examination

Clinical examination and testing were performed, and the following data were collected: patient age (at the time of surgery), sex, tumor diameter (measured by B-scan ultrasonography), tumor thickness (measured by A- or B-scan ultrasonography). Patients were assessed for the presence or absence of exudative retinal detachment (RD), lipofuscin, drusen, retinal pigment epithelial fibrosis and epithelial atrophy, and low internal reflectivity (determined using A- or B-scan). Tumors were staged according to the AJCC classification.[1, 2]

Vitreous sample collection

Liquid vitreous biopsies were collected from two groups: eyes from control subjects (n = 3) with epiretinal membranes (ERMs), and eyes from test subjects (n = 8) with UM. Vitreous biopsies were collected in the operating room to ensure timely sample processing and storage. In eyes undergoing I-125 plaque brachytherapy for UM treatment or control eyes with ERM, a standard 3 port pars plana vitrectomy setup was used with a single-step transconjunctival 27-gauge trocar cannular system (Alcon Laboratories Inc, Fort Worth, TX). Undiluted 0.5 to 1.0cc sample of the vitreous was manually aspirated into a 3-cc syringe. Vitreous biopsies were collected prior to placement of the active I-125 plaque and before tumor biopsy. No vitreous biopsy complications (e.g., retinal tears, detachments, or hemorrhages) were noted. For UM eyes undergoing enucleation, after the globe was removed an 18-gauge needle was inserted through the pars plana in the quadrant opposite the bulk of the tumor and 1-2cc of vitreous was removed in the operating room prior to formalin fixation. Trans-scleral tumor biopsy (without vitreous violation) was performed prior to vitreous collection. Vitreous samples were immediately centrifuged in the operating room at 15,000 x g for 5 minutes at room temperature to remove impurities and then finally stored at -80˚C, as previously described.[3]

Tumor biopsy

The GEP profile of the tumor samples was determined as previously described.[4, 5] Briefly, a fine-needle aspiration (FNA) or vitrectomy assisted biopsy of the tumor was performed at the time of I-125 plaque brachytherapy or at the time of enucleation.[6, 7] Tumor samples underwent RNA extraction followed by reverse transcription to generate cDNA and by analysis by real-time quantitative PCR (Decision Dx-UM, Castle Biosciences).[4, 5] The PRAME status of the tumor samples was determined by measuring *PRAME* mRNA expression on an Illumina HT-12v4 chip using probe ILMN_1700031 as described previously (Decision Dx-UM PRAME, Castle Biosciences).[8]

Multiplex ELISA array

Vitreous samples were analyzed using a quantitative multiplex ELISA array that concurrently measured the

levels of 1,000 proteins (described below). Twenty proteins were selected from the training dataset for prospective vitreous (n = 22) and plasma (n = 13) analysis in separate cohort of patients. Vitreous cytokine signaling proteins were measured using the Human Kiloplex Array Q1 (RayBio, Norcross, GA) per the manufacturers protocol. This array concurrently detected and processed 1,000 human proteins. Vitreous samples from the training dataset cohort were diluted in phosphate-buffered saline (PBS; pH 7.4) to a final volume of 1.5 mL. For the validation study, undiluted vitreous or plasma (130 μL) was applied to the custom array chip. First, the array chips were incubated with sample diluents for 30 minutes at room temperature to act as a block. Vitreous (four technical replicates per sample) was added to the wells of the array and incubated overnight at 4˚C. A standard protein dilution was added to the wells of the array to determine protein concentrations. For signal detection, 80 μL of Cy3-streptavidin was added to each well, rinsed and visualized by laser scanner. The RayBio® Analysis Tool (RayBio®, Norcross, GA) was used for protein classification. Final protein concentrations (in pg/mL) were corrected for sample dilution.

Bioinformatic analysis

Results from the separate training datasets were saved in Excel as .txt format and were uploaded into the Partek Genomics Suite 6.5 software package. The data was normalized to log base 2 and compared using 1-way ANOVA analysis as previously described.[3, 9, 10] All proteins with non-significant ($p>0.05$) changes were eliminated from the table. The significant values were mapped using the 'cluster based on significant genes' visualization function with the standardization option chosen. Principal component analysis was performed using Qlucore Omics Explorer 3.2 software. Since the multiplex ELISA does not represent the entire vitreous proteome, we used an overrepresentation analysis (ORA)-based method with the 1,000 proteins on the array as the reference protein list.[11-13] Enriched pathways were detected by searching the Reactome[14], PANTHER[15], KEGG[16], and WikiPathways[17] databases using the WebGestalt toolkit.[18] Differentially expressed proteins ($p < 0.05$) in UM vitreous were also analyzed using the core analysis function in Ingenuity Pathway Analysis (IPA).[13] Pathway analysis was performed using an ORA method followed by Benjamini-Hochberg multiple test adjustments.[12] The full list of 1,000 proteins represented on the Human Kiloplex Array Q1 (https://www.raybiotech.com/human-kiloplex/) was used as the reference protein list.

Statistical analysis

Results from the verification datasets were analyzed using Grubb's maximum normalized residual test to detect the presence of outliers (Alpha = 0.05).[19, 20] Results were then analyzed using Student's pairwise t-test or 1-way ANOVA followed by Tukey's multiple comparison test (significant p-value < 0.05).

Case Series

Case 1 – A 67-year-old male presented for consultation for a uveal melanoma in the left eye measuring 16 x 13.66 x 5.8 mm. The patient reported a six-month history of blurry vision with waves of light. He was seen by a local eye doctor who noted an ocular lesion and retinal detachment prior to referral. He had no prior history of eye surgery or eye problems. Left eye enucleation with orbital implant was subsequently performed and a biopsy was sent for genetic testing. The biopsy revealed both spindle and epithelioid cells (G2) with focal emissary vein and inner scleral invasion without extraocular tumor extension (EOE). The tumor was found to be gene expression profile (GEP) Class 1B, or intermediate risk disomy 3, and PRAME positive [21-23]. The American Joint Committee on Cancer (AJCC) classification was T3a with an anatomic stage III (T3, N0, M0) [1, 2].

Case 2 – A 30-year-old female with a family history of cancer (BAP1 deletion) presented for a second opinion regarding a retinal lesion in the left eye found to be uveal melanoma measuring 6 x 7 x 2.5 mm. The patient had regular eye exams for the past 4 years but, by her account, had never been dilated at these exams. She noted a two-month history of worsening vision of the left eye. She was seen by an optometrist and subsequently a retina specialist and found to have a peripapillary elevated lesion of the left eye. She has a strong family history of cancer, including mesothelioma in her grandmother, and breast and lung cancer in her mother. She had a breast and thyroid thermography test performed which showed some suspicious activity in her thyroid but no concerning breast activity. Left eye enucleation with orbital implant was subsequently performed and a biopsy was sent for genetic testing. The biopsy revealed both spindle A and spindle B cells (G1). The tumor was found to be GEP Class 1A, or low risk disomy 3, PRAME positive. The AJCC classification was T1a with an anatomic stage I (T1a, N0, M0).

Case 3 – A 80-year-old male with a history of parkinsonism and ANS dysfunction presented for a consultation for retinal detachment in the left eye and was found to have uveal melanoma measuring 12.7 x 11.22 x 4.75 mm. The patient had undergone cataract surgery in both eyes six months prior and had blurry vision in the left eye since that time. His vision had been stably poor with no flashes or floaters and very mild intermittent pain. Left eye enucleation with orbital implant was performed and a biopsy was sent for genetic testing. The biopsy revealed >90% epithelioid cells (G3). The tumor was found to be GEP Class 2, PRAME positive. The AJCC classification was T2a with an anatomic stage IIA (T2a, N0, M0).

Case 4 – A 47-year-old male presented for a consultation for a mass in the right eye found to be an amelanotic uveal melanoma measuring 11.48 x 11.75 x 7.5 mm. The patient described a subtle decrease in vision in the right eye three weeks prior to presentation with a curtain veil noted. He had been seen 9-months prior by the referring physician with a normal posterior segment exam. Right eye I-125 plaque brachytherapy was performed with a vitrectomy assisted-FNA biopsy and injection of bevacizumab at the time of plaque insertion. The biopsy revealed predominantly epithelioid cells (G3) and the SOX10 immunohistochemistry stain was positive in lesional cells. The tumor was found to be GEP Class 1A, PRAME negative. The AJCC classification

was T2a with an anatomic stage IIA (T2a, NX, MX).

Case 5 – A 57-year-old diabetic male presented on for a consultation for ocular melanoma in the right eye measuring 11 mm x 9 mm x 3.99 mm. The patient had no vision changes and had initially been sent for a diabetic eye exam when a choroidal lesion was noted in the right eye. Right eye I-125 plaque brachytherapy was performed with a vitrectomy assisted-FNA biopsy at the time of insertion. The tumor was found to be GEP Class 1A, PRAME negative. The pathology did not result. The AJCC classification was T2a with an anatomic stage IIA (T2a, NX, MX).

Case 6 – A 57-year-old male presented for a consultation for ciliary body mass in the left eye found to be uveal melanoma measuring 21.8 x 16.66 x 12.74 mm. The patient noted a two-year history of vision changes which worsened over the past 2 months with no other prior vision problems. He had been trying unsuccessfully for 1-2 months to obtain a functional prescription for eyeglass. He denied pain but noted a pressure-like sensation. He reported darkness in his peripheral field of vision as well as photopsias for the past 3 weeks with dramatic changes leading up to his presentation. The patient's last dilated fundus examination had been performed one year prior to the noticed growth in his eyes by his doctor. Following this, he was seen by two referring physicians who reported large ciliary body mass lesions. Ophthalmic imaging showed the right eye with flat dark choroidal nevus and left eye with large temporal mass with inferior retinal detachment. Left eye enucleation with orbital implant was performed and a biopsy was sent for genetic testing. The biopsy revealed mixed spindle and epithelioid cell types (G2) confined to the uveal tract and abutting the ciliary body with negative surgical margins. The tumor was found to be GEP Class 2, PRAME positive. There were no metastases noted at the time of the procedure, but liver metastases have since been noted. The AJCC classification was T4b with an anatomic stage IIB (T3a, N0, M0) at the time of procedure and IV (T4b, N1, M1) following.

Case 7 – A 35-year-old female with a history of Henoch–Schönlein purpura presented for a consultation for possible subretinal fluid in left eye and was found to have uveal melanoma measuring 15.6 x 15.9 x 7.3 mm. The patient noted several years of an intermittent "heat wave" pattern in the left eye with a 1-month history of peripheral vision changes. She experienced rare floaters but no photopsias, eye pain, photophobia nor trauma. She denied prior eye surgery, laser procedures, or intravitreal injections. Left eye I-125 plaque brachytherapy was performed with a vitrectomy assisted-FNA biopsy at the time of insertion. The tumor was found to be GEP Class 1B, PRAME negative. The pathology did not result. The AJCC classification was T3a with an anatomic stage IIB (T3a, N0, M0).

Case 8 – A 58-year-old male with a recent diagnosis of prostate cancer (the day prior to presentation) presented for a consultation for choroidal lesion to the left eye found to be uveal melanoma measuring 15.6 x 14.5 x 7.3 mm. The patient awoke two weeks prior and noticed that vision in the left eye was significantly decreased with an increase in floaters. He denied recent trauma or eye pain. He immediately saw an

optometrist who noted "bleeding in the eye" and suspected a retinal detachment possibly with a growth noted. The patient saw another referring patient on the same day who performed an ultrasound which revealed possible melanoma with a vitreous hemorrhage. He described his vision in the left eye as looking through fog and shadow with an inability to read or focus. The patient's mother has glaucoma and his older brother was previously diagnosed with prostate cancer as well. Right eye I-125 plaque brachytherapy was performed with a vitrectomy assisted-FNA biopsy and injection of bevacizumab at the time of plaque insertion. Biopsy revealed a primarily epithelioid morphology (G3). The tumor was found to be GEP Class 2, PRAME negative. The AJCC classification was T3a with an anatomic stage IIB (T3a, N0, M0).

Supplemental References

1. Force AOOT: **International Validation of the American Joint Committee on Cancer's 7th Edition Classification of Uveal Melanoma**. *JAMA Ophthalmol* 2015, **133**(4):376-383.
2. Mellen PL, Morton SJ, Shields CL: **American joint committee on cancer staging of uveal melanoma**. *Oman J Ophthalmol* 2013, **6**(2):116-118.
3. Velez G, Roybal CN, Colgan D, Tsang SH, Bassuk AG, Mahajan VB: **Precision Medicine: Personalized Proteomics for the Diagnosis and Treatment of Idiopathic Inflammatory Disease**. *JAMA Ophthalmol* 2016, **134**(4):444-448.
4. Cai L, Paez-Escamilla M, Walter SD, Tarlan B, Decatur CL, Perez BM, Harbour JW: **Gene Expression Profiling and PRAME Status Versus Tumor-Node-Metastasis Staging for Prognostication in Uveal Melanoma**. *Am J Ophthalmol* 2018, **195**:154-160.
5. Harbour JW, Paez-Escamilla M, Cai L, Walter SD, Augsburger JJ, Correa ZM: **Are Risk Factors for Growth of Choroidal Nevi Associated With Malignant Transformation? Assessment With a Validated Genomic Biomarker**. *Am J Ophthalmol* 2019, **197**:168-179.
6. Grewal DS, Cummings TJ, Mruthyunjaya P: **Outcomes of 27-Gauge Vitrectomy-Assisted Choroidal and Subretinal Biopsy**. *Ophthalmic Surg Lasers Imaging Retina* 2017, **48**(5):406-415.
7. Finn AP, Materin MA, Mruthyunjaya P: **CHOROIDAL TUMOR BIOPSY: A Review of the Current State and a Glance Into Future Techniques**. *Retina* 2018, **38 Suppl 1**:S79-S87.
8. Gezgin G, Luk SJ, Cao J, Dogrusoz M, van der Steen DM, Hagedoorn RS, Krijgsman D, van der Velden PA, Field MG, Luyten GPM *et al*: **PRAME as a Potential Target for Immunotherapy in Metastatic Uveal Melanoma**. *JAMA Ophthalmol* 2017, **135**(6):541-549.
9. Velez G, Bassuk AG, Colgan D, Tsang SH, Mahajan VB: **Therapeutic drug repositioning using personalized proteomics of liquid biopsies**. *JCI Insight* 2017, **2**(24).
10. Roybal CN, Velez G, Toral MA, Tsang SH, Bassuk AG, Mahajan VB: **Personalized Proteomics in Proliferative Vitreoretinopathy Implicate Hematopoietic Cell Recruitment and mTOR as a Therapeutic Target**. *Am J Ophthalmol* 2018, **186**:152-163.
11. Zhang B, Kirov S, Snoddy J: **WebGestalt: an integrated system for exploring gene sets in various biological contexts**. *Nucleic Acids Res* 2005, **33**(Web Server issue):W741-748.
12. Garcia-Campos MA, Espinal-Enriquez J, Hernandez-Lemus E: **Pathway Analysis: State of the Art**. *Front Physiol* 2015, **6**:383.
13. Kramer A, Green J, Pollard J, Jr., Tugendreich S: **Causal analysis approaches in Ingenuity Pathway Analysis**. *Bioinformatics* 2014, **30**(4):523-530.
14. Jassal B, Matthews L, Viteri G, Gong C, Lorente P, Fabregat A, Sidiropoulos K, Cook J, Gillespie M, Haw R *et al*: **The reactome pathway knowledgebase**. *Nucleic Acids Res* 2020, **48**(D1):D498-D503.
15. Mi H, Thomas P: **PANTHER pathway: an ontology-based pathway database coupled with data analysis tools**. *Methods Mol Biol* 2009, **563**:123-140.
16. Kanehisa M, Goto S: **KEGG: kyoto encyclopedia of genes and genomes**. *Nucleic Acids Res* 2000, **28**(1):27-30.

17. Slenter DN, Kutmon M, Hanspers K, Riutta A, Windsor J, Nunes N, Melius J, Cirillo E, Coort SL, Digles D *et al*: **WikiPathways: a multifaceted pathway database bridging metabolomics to other omics research**. *Nucleic Acids Res* 2018, **46**(D1):D661-D667.
18. Liao Y, Wang J, Jaehnig EJ, Shi Z, Zhang B: **WebGestalt 2019: gene set analysis toolkit with revamped UIs and APIs**. *Nucleic Acids Res* 2019, **47**(W1):W199-W205.
19. Horwitz W: **IUPAC Protocol for the design, conduct and interpretation of method performance studies**. *Pure Appl Chem* 1995, **67**(2):331-343.
20. Ellison SLR, Barwick VJ, Farrant TJD: **Practical Statistics for the Analytical Scientist**: RSC Publishing; 2009.
21. Field MG, Decatur CL, Kurtenbach S, Gezgin G, van der Velden PA, Jager MJ, Kozak KN, Harbour JW: **PRAME as an Independent Biomarker for Metastasis in Uveal Melanoma**. *Clin Cancer Res* 2016, **22**(5):1234-1242.
22. Onken MD, Worley LA, Char DH, Augsburger JJ, Correa ZM, Nudleman E, Aaberg TM, Jr., Altaweel MM, Bardenstein DS, Finger PT *et al*: **Collaborative Ocular Oncology Group report number 1: prospective validation of a multi-gene prognostic assay in uveal melanoma**. *Ophthalmology* 2012, **119**(8):1596-1603.
23. Onken MD, Worley LA, Tuscan MD, Harbour JW: **An accurate, clinically feasible multi-gene expression assay for predicting metastasis in uveal melanoma**. *J Mol Diagn* 2010, **12**(4):461-468.
24. Song J, Merbs SL, Sokoll LJ, Chan DW, Zhang Z: **A multiplex immunoassay of serum biomarkers for the detection of uveal melanoma**. *Clin Proteomics* 2019, **16**:10.
25. Shi XY, Li Q, Wei WB, Tao LM: **Peptidome profiling of human serum of uveal melanoma patients based on magnetic bead fractionation and mass spectrometry**. *Int J Ophthalmol* 2017, **10**(6):939-947.
26. Linge A, Kennedy S, O'Flynn D, Beatty S, Moriarty P, Henry M, Clynes M, Larkin A, Meleady P: **Differential expression of fourteen proteins between uveal melanoma from patients who subsequently developed distant metastases versus those who did Not**. *Invest Ophthalmol Vis Sci* 2012, **53**(8):4634-4643.
27. Pardo M, Garcia A, Thomas B, Pineiro A, Akoulitchev A, Dwek RA, Zitzmann N: **Proteome analysis of a human uveal melanoma primary cell culture by 2-DE and MS**. *Proteomics* 2005, **5**(18):4980-4993.
28. Pardo M, Garcia A, Antrobus R, Blanco MJ, Dwek RA, Zitzmann N: **Biomarker discovery from uveal melanoma secretomes: identification of gp100 and cathepsin D in patient serum**. *J Proteome Res* 2007, **6**(7):2802-2811.
29. Wierenga APA, Cao J, Mouthaan H, van Weeghel C, Verdijk RM, van Duinen SG, Kroes WGM, Dogrusoz M, Marinkovic M, van der Burg SSH *et al*: **Aqueous Humor Biomarkers Identify Three Prognostic Groups in Uveal Melanoma**. *Invest Ophthalmol Vis Sci* 2019, **60**(14):4740-4747.
30. Crabb JW, Hu B, Crabb JS, Triozzi P, Saunthararajah Y, Tubbs R, Singh AD: **iTRAQ Quantitative Proteomic Comparison of Metastatic and Non-Metastatic Uveal Melanoma Tumors**. *PLoS One* 2015, **10**(8):e0135543.

31. Zuidervaart W, Hensbergen PJ, Wong MC, Deelder AM, Tensen CP, Jager MJ, Gruis NA: **Proteomic analysis of uveal melanoma reveals novel potential markers involved in tumor progression**. *Invest Ophthalmol Vis Sci* 2006, **47**(3):786-793.

Table S1. Proteomic studies on uveal melanoma using human tissue. *Abbreviations: LC-MS/MS = liquid chromatography-tandem mass spectrometry; MALDI-TOF = matrix-assisted laser desorption/ionization time-of-flight; 2D-DIGE = two-dimensional difference gel electrophoresis; iTRAQ = isobaric tags for relative and absolute quantitation.

Study	Platform	Tissue	UM Sample Size	Differentially Expressed Proteins
Velez, et al. (Current study)	Multiplex immunoassay (1,000 proteins)	Vitreous and Plasma	19	HGF, HGFR/c-Met, SCFR/c-Kit, Autotaxin, Arginase-1, and KLK7
Song, et al.[24]	Multiplex immunoassay (7 proteins)	Serum	48	HSP7 and Osteopontin, MIA
Shi, et al.[25]	Magnetic bead capture, MALDI-TOF MS	Serum	18	Fibrinogen
Linge, et al.[26]	2D-DIGE, LC-MS/MS	Primary Tumors	25	FABP3, TPI1, PDIA3, VIM, SELENBP1, ENO1, CAPZA1, ERP29, PARK7
Pardo et al.[27]	2D-DIGE, LC-MS/MS	Primary Tumor Cell Culture	1	Ezrin, HSP90, S100, PARK7, VIM, Cytokeratins 8 and 18
Pardo et al.[28]	2D-DIGE, LC-MS/MS	Cell Culture Secretomes	1	Cathepsin D, Syntenin, gp100, HGFR, IGF-2R
Wierenga, et al.[29]	Multiplex immunoassay (92 proteins)	Aqueous Humor	90	Galectin-9, TRAIL, Fas-L, ADA, CD244, CD40, MCP-3, PD-L1
Crabb, et al.[30]	iTRAQ	Primary Tumors	15	Collagen alpha-3 (VI), HSP27, FABP3, β-hexosaminidase
Zuidervaart et al.[31]	2D-DIGE, LC-MS/MS	Primary Tumor and Metastases	1	HSP27, Galectin-1, Cofilin, GST, β-hexosaminidase

Table S2. Patient demographics for training dataset

Case	Sex	Age	Eye	Tumor Size (mm)	PRAME Status	GEP Class	AJCC Stage
1	M	67	OS	16 x 13.7 x 5.8	Positive	1B	IIIA
2	F	30	OS	6.0 x 7.0 x 2.5	Positive	1A	IA
3	M	80	OS	12.7 x 11.2 x 4.8	Positive	2	IIA
4	M	47	OD	11.5 x 11.8 x 7.5	Negative	1A	IIA
5	M	57	OD	11.0 x 9.0 x 3.9	Negative	1A	IIA
6	M	57	OS	21.8 x 16.7 x 12.8	Positive	2	IIB
7	F	35	OS	15.6 x 15.9 x 7.3	Negative	1B	IIB
8	M	58	OS	15.6 x 14.5 x 7.3	Negative	2	IIB

Table S3. Differentially expressed ELISA proteins based on GEP class

Protein	p-value (GEP)	p-value (Class 1 vs. Control)	Fold-Change (Class 1 vs. Control)	p-value (Class 2 vs. Control)	Fold-Change (Class 2 vs. Control)	p-value (Class 2 vs. Class 1)	Fold-Change (Class 2 vs. Class 1)
FABP1	1.41E-11	3.15E-12	-2.54E+07	6.52E-12	-2.54E+07	1	1
DNMT3A	4.41E-09	1	1	2.04E-09	9.26E+06	9.89E-10	9.26E+06
Siglec-6	8.36E-08	2.20E-08	107289	2.97E-08	221112	0.130566	2.0609
Kallikrein 7	2.30E-07	5.17E-08	-94797	1.06E-07	-94797	1	1
OMgp	4.51E-07	4.82E-06	-1107.98	0.0953236	3.3153	1.66E-06	3673.29
Arginase 1	1.12E-06	1	1	5.14E-07	4.31E+06	2.52E-07	4.31E+06
OSM	1.13E-06	5.35E-07	15054.2	2.47E-07	153545	0.004188	10.1995
MEF2C	1.47E-06	1	1	6.73E-07	1.43E+06	3.30E-07	1.43E+06
Common beta Chain	3.42E-06	1	1	1.56E-06	694048	7.70E-07	694048
Granzyme A	4.74E-06	1	1	2.17E-06	68885.7	1.07E-06	68885.7
Sirtuin 1	5.01E-06	1	1	2.29E-06	2.52E+06	1.13E-06	2.52E+06
IL-8	1.21E-05	1	1	5.51E-06	6929.82	2.74E-06	6929.82
CD27	4.64E-05	0.00356218	97.493	2.72E-05	88155.8	0.00036983	904.227
MIS RII	0.00014871	1	1	6.64E-05	186602	3.39E-05	186602
MIP-1a	0.000181	0.00391934	46.899	0.00019982	1261.94	0.00858141	26.9076
HB-EGF	0.00018311	0.120785	-1.45494	0.00731298	2.41054	0.00059402	3.50718
HO-1	0.00087724	0.367159	2.83411	0.0002973	2814.43	0.00037225	993.056
IL-16	0.00099892	0.13305	11.6905	0.00117358	4641.8	0.00437172	397.057
DAPP1	0.00111247	1	1	0.00048384	11893.8	0.0002569	11893.8
GM-CSF Ra	0.00206575	0.00033057	-441737	0.00251104	-26036.9	0.199065	16.9658
MMP-13	0.0021909	0.0120799	8.67041	0.00034532	100.509	0.00660998	11.5922
Cathepsin S	0.00224408	0.0460366	2.12959	0.00073699	7.19839	0.00589612	3.38018
S100A13	0.00288434	0.897934	-1.13859	0.0028471	128.51	0.00138508	146.32
TGFa	0.00368794	0.557453	2.29556	0.00493699	422.505	0.00616863	184.053
GPR56	0.00371868	0.0490115	3.54843	0.00066421	30.7567	0.00483872	8.66767
CES2	0.00384001	0.248021	18.7868	0.00131028	611555	0.00292267	32552.4
SCF R	0.0048514	0.0143891	9.89105	0.00080831	82.3164	0.0202408	8.32231
PIGF	0.00496676	0.252041	8.89605	0.00170705	14228.1	0.00396761	1599.38
TRAIL R3	0.00558777	0.179859	2.26772	0.00175767	19.8055	0.00557742	8.73366
TAFA1	0.0056455	0.00587704	-7447.92	0.768706	2.17015	0.00383058	16163.1
Galectin-9	0.00573014	0.366733	1.65751	0.00317524	12.8527	0.00581489	7.75421
Cystatin B	0.00617715	0.0452452	3.71738	0.00151394	20.3233	0.0162137	5.46709
Angiogenin	0.00653827	0.156838	1.25705	0.00153027	2.23333	0.0052991	1.77665
EG-VEGF	0.00673961	0.270493	12.5327	0.00231959	55330.8	0.00538301	4414.91
Integrin alpha 5	0.0072314	0.00948558	-78783.1	0.660244	5.06244	0.00488426	398835
HGF R	0.00753933	0.117975	2.73008	0.00138483	24.4099	0.00600158	8.94111
PDGF Rb	0.00812138	0.201648	37.6118	0.00249033	504296	0.00773788	13407.9
Siglec-9	0.00850629	0.670143	-2.6714	0.0629871	224.887	0.0231855	600.762
ROBO3	0.00856288	0.0102727	-7.56693	0.371439	1.85178	0.00267123	14.0123
CHMP2B	0.00862391	0.00797598	170223	0.00275923	1.37E+07	0.223205	80.4428

GDF-15	0.00893597	0.320003	1.85641	0.00260876	18.6033	0.00527517	10.0211
PDGF-AA	0.00895789	0.0479921	3.1195	0.00193015	12.6881	0.0213928	4.06735
Ephrin-B3	0.00959977	0.30057	25.4737	0.0219542	12367.2	0.0701194	485.49
FUCA1	0.00962622	0.900785	1.04426	0.00715577	4.03284	0.00498028	3.8619
Ferritin	0.00967032	0.103758	6.00668	0.00205892	158.317	0.0112645	26.3569
ANGPTL4	0.00994493	0.0153275	19.3751	0.00284933	102.16	0.116922	5.27275

Table S4. Differentially expressed ELISA proteins based on PRAME status

Protein	p-value (PRAME)	p-value (Positive vs. Control)	Fold-Change (Positive vs. Control)	p-value (Negative vs. Control)	Fold-Change (Negative vs. Control)	p-value (Positive vs. Negative)	Fold-Change (Positive vs. Negative)
FABP1	1.51E-13	1.08E-13	-2.54E+07	1.08E-13	-2.54E+07	1	1
Desmoglein -3	1.10E-09	1.44E-09	64708.6	1	1	7.83E-10	64708.6
Kallikrein 7	9.91E-09	7.07E-09	-94797	7.07E-09	-94797	1	1
Siglec-6	2.54E-08	1.84E-08	132115	1.78E-08	138910	0.921528	-1.05143
EphB6	1.91E-06	2.41E-06	392025	1	1	1.34E-06	392025
OSM	1.62E-05	7.49E-06	62026.4	1.92E-05	16662.3	0.227161	3.72255
GM-CSF Ra	0.0005392	0.0002212	-441737	0.00071913	-52842.5	0.29521	-8.35951
PP	0.00094297	0.00031037	12.0526	0.0024403	6.01525	0.10617	2.00367
Furin	0.0012448	0.893675	-1.02755	0.00118763	-2.62709	0.00087648	2.55667
Podoplanin	0.00172609	0.0556448	261.629	0.0191317	-1448.94	0.00052441	379086
SOX15	0.00312145	0.0126317	1135.1	0.194913	-22.4349	0.00107516	25465.7
GFAP	0.00339276	0.00162764	2.5416	0.288317	1.25578	0.00521598	2.02393
ENPP-7	0.00372146	0.00191911	27149.8	0.356042	9.08256	0.00496639	2989.22
ROBO4	0.00420071	0.135063	203.646	0.0239078	-7283.9	0.00135808	1.48E+06
Follistatin-like 1	0.00457415	0.00334798	7.08017	0.714967	1.19699	0.00373321	5.91498
GATA-4	0.00617188	0.0019966	9.61E+06	0.0170277	45255.8	0.143762	212.385
BAMBI	0.00653629	0.00290163	4.68256	0.27579	1.53351	0.010882	3.05349
SLITRK5	0.00655776	0.0035087	8.23222	0.438136	1.52357	0.0077185	5.40324
SorCS3	0.00666277	0.00322331	3.27E+06	0.348879	36.5714	0.00930123	89424.4
HAO-1	0.00679907	0.0127883	23347.2	0.00236015	980292	0.236209	-41.9875
GFR alpha-2	0.00694925	0.00724513	2.59236	0.909136	-1.03189	0.00401526	2.67502
Semaphorin 7A	0.00758048	0.341038	1.30654	0.0195368	-2.15784	0.00284354	2.8193
IL-2	0.00764944	0.00274229	-5367.21	0.0126477	-626.183	0.282401	-8.57132
TROY	0.00804147	0.467769	-8.92378	0.00435513	-79606.3	0.0090638	8920.69
TPP1	0.00831942	0.315778	1.22212	0.0229781	-1.69214	0.00305067	2.068
PDX-1	0.00837276	0.00304391	1.93E+06	0.0131292	57747	0.304741	33.3681
CHMP2B	0.00845654	0.00307648	1.00E+07	0.013223	202365	0.3061	49.5443
CrkL	0.00913102	0.00334335	88382.8	0.0139186	5796.01	0.318142	15.2489
Glypican 1	0.00983008	0.0124924	6.10165	0.729242	-1.22411	0.00487412	7.46911

Table S5. Vitreous biomarkers for verification

Protein	UniProt	Full Protein Name	Patient Group
FABP1	P07148	Fatty acid-binding protein 1	Control
GM-CSF Ra	P15509	Granulocyte-macrophage colony-stimulating factor receptor	Control
KLK7	P49862	Kallikrein 7	Control
SIGL6	O43699	Sialic acid-binding Ig-like lectin 6	CM (All Groups)
MYC	P01106	Myc proto-oncogene protein	CM (All Groups)
OSM	P13725	Oncostatin-M	CM (All Groups)
SCFR/KIT	P10721	Stem cell growth factor receptor Kit (Gleevac)	CM (All Groups)
CSF2RB	P32927	Common beta chain	GEP Class 2
c-MET/HGFR	P08581	Hepatocyte growth factor receptor (c-MET)	GEP Class 2
SIR1	Q96EB6	Sirtuin-1	GEP Class 2
GRAA	P12544	Granzyme A	GEP Class 2
MEF2C	Q06413	Myocyte-specific enhancer factor 2C	GEP Class 2
ARGI1	P05089	Arginase-1	GEP Class 2
FAS L	P48023	Fas ligand	GEP Class 2
PP/PAHO	P01298	Pancreatic prohormone	GEP Class 2
DNMT3A	Q9Y6K1	DNA (cytosine-5)-methyltransferase 3A	GEP Class 2
DSG3	P32926	Desmoglein-3	PRAME Positive
ENPP2	Q13822	Autotaxin	PRAME Positive
LEG9	O00182	Galectin-9	PRAME Positive
HGF	P14210	Hepatocyte growth factor	PRAME Positive

Table S6. Patient demographics for validation study

Case	Sex	Age	Eye	Diagnosis	Sample Type	PRAME Status	GEP Class	AJCC Stage
1	M	67	OS	Epiretinal Membrane	Vitreous	N/A	N/A	N/A
2	M	66	OS	Epiretinal Membrane	Vitreous	N/A	N/A	N/A
3	M	89	OS	Epiretinal Membrane	Vitreous	N/A	N/A	N/A
4	F	70	OS	Epiretinal Membrane	Vitreous	N/A	N/A	N/A
5	M	63	OS	Epiretinal Membrane	Vitreous	N/A	N/A	N/A
6	M	75	OS	Epiretinal Membrane	Vitreous	N/A	N/A	N/A
7	M	75	OS	Epiretinal Membrane	Vitreous	N/A	N/A	N/A
8	F	81	OD	Epiretinal Membrane	Vitreous	N/A	N/A	N/A
9	F	61	OS	Macular Hole	Vitreous	N/A	N/A	N/A
10	F	67	OD	Macular Hole	Vitreous	N/A	N/A	N/A
11	M	76	OS	Retained Lens Fragment	Vitreous	N/A	N/A	N/A
12	M	72	OD	Uveal Melanoma	Vitreous	Negative	2	I
13	M	80	OD	Uveal Melanoma	Vitreous	Positive	2	IV
14	F	32	OS	Uveal Melanoma	Vitreous	Negative	1A	IIB
15	M	41	OS	Uveal Melanoma	Vitreous	Positive	2	IIIB
16	F	62	OS	Uveal Melanoma	Vitreous	Negative	1A	I
17	F	70	OD	Uveal Melanoma	Vitreous	Negative	1B	IIA
18	M	66	OD	Uveal Melanoma	Vitreous	Negative	1B	IIA
19	F	30	OS	Uveal Melanoma	Vitreous	Positive	1A	I
20	F	60	OD	Uveal Melanoma	Vitreous	Positive	1A	IIIA
21	M	50	OD	Uveal Melanoma	Vitreous	Positive	1B	IIIA
22	M	49	OD	Uveal Melanoma	Vitreous	Negative	1A	I
23	F	30	OS	Uveal Melanoma	Plasma	Positive	1A	I
24	M	80	OD	Uveal Melanoma	Plasma	Positive	2	IV
25	M	49	OD	Uveal Melanoma	Plasma	Negative	2	I
26	F	70	OD	Uveal Melanoma	Plasma	Negative	1B	IIA
27	F	32	OS	Uveal Melanoma	Plasma	Negative	1A	IIB
28	F	62	OS	Uveal Melanoma	Plasma	Negative	1A	I
29	M	72	OD	Uveal Melanoma	Plasma	Negative	2	I
30	M	66	OD	Uveal Melanoma	Plasma	Negative	1B	IIA
31	M	72	OS	Epiretinal Membrane	Plasma	N/A	N/A	N/A
32	M	77	OS	Choroidal Nevus	Plasma	N/A	N/A	N/A
33	F	68	OD	Epiretinal Membrane	Plasma	N/A	N/A	N/A
34	M	55	OD	Choroidal Nevus	Plasma	N/A	N/A	N/A
35	F	56	OS	Macular Hole	Plasma	N/A	N/A	N/A

Table S7. Vitreous biomarker verification: Expression data of is represented as protein concentration (pg/mL) and displayed as mean ± SEM. Data were analyzed by Tukey's multiple comparison test or by Student's pairwise t-test, where appropriate (Alpha = 0.05).

	Vitreous Protein Expression					
	Control (pg/mL)		Uveal Melanoma (pg/mL)		Statistics	
Protein	Mean	SEM	Mean	SEM	p-value	Significance
ARGI1	5.2	2.8	36.2	35	0.383	No
βc	300	21	266	56	0.590	No
DNMT3A	812	220	953	120	0.583	No
ENPP-2	82,700	3,200	70,000	2,700	0.007	Yes
GRAAA	20.9	2.6	42.7	22	0.357	No
KLK7	1,250	150	549	110	0.001	Yes
MEF2C	191	46	163	59	0.714	No
c-Myc	367	45	211	52	0.033	Yes
PP	11,200	930	7,800	880	0.016	Yes
SIGL6	0	0	12.4	11	0.309	No
SIRT1	24.8	11	54.7	39	0.490	No
DSG3	0	0	1.26	1.3	0.353	No
FABP1	185	96	30,900	27,000	0.294	No
FASL	0.11	0.06	3.63	2.3	0.160	No
LEG9	527	88	549	75	0.847	No
GM-CSF R	0	0	49.1	49	0.353	No
HGF	1,672	230	2,920	510	0.044	Yes
HGFR/c-MET	5,830	830	7,760	980	0.149	No
OSM	0.84	0.41	4.87	2.5	0.148	No
SCFR/c-Kit	135	32	1,140	370	0.011	Yes

Table S8. Plasma biomarker verification: Expression data of is represented as protein concentration (pg/mL) and displayed as mean ± SEM. Data were analyzed by Tukey's multiple comparison test or by Student's pairwise t-test, where appropriate (Alpha = 0.05).

	Plasma Protein Expression					
	Control (pg/mL)		Uveal Melanoma (pg/mL)		Statistics	
Protein	Mean	SEM	Mean	SEM	p-value	Significance
ARGI1	874	120	1,790	300	0.042	Yes
βc	3,370	850	1,030	260	0.008	Yes
DNMT3A	1,630	460	1,420	410	0.741	No
ENPP-2	73,500	5,400	96,200	7,200	0.046	Yes
GRAAA	443	95	269	77	0.187	No
KLK7	6,430	2,100	3,410	730	0.131	No
MEF2C	1,820	1,000	473	190	0.124	No
c-Myc	7,360	5,400	834	310	0.145	No
PP	38,900	13,000	16,800	4,900	0.095	No
SIGL6	257	63	155	18	0.083	No
SIRT1	2,640	2,000	737	280	0.244	No
DSG3	98.6	55	58.3	15	0.402	No
FABP1	743,000	460,000	68,500	35,000	0.082	No
FASL	258	130	63.5	38	0.112	No
LEG9	1,890	470	1,130	410	0.262	No
GM-CSF R	1,990	1,330	429	210	0.169	No
HGF	231	99	137	17	0.259	No
HGFR/c-MET	13,500	1,450	12,100	490	0.280	No
OSM	665	540	52.9	24	0.167	No
SCFR/c-Kit	10,100	2,800	5,090	890	0.064	No

Fig. S1. Vitreous biomarker levels in training dataset: Protein expression levels for **(A)** Fatty acid binding protein 1 (FABP1), **(B)** Granulocyte-macrophage colony-stimulating factor (GM-CSF Ra), **(C)** Kallikrein 7 (KLK7), **(D)** Oncostatin M (OSM), **(E)** c-Myc, **(F)** Siglec-6, **(G)** Stem cell factor receptor (SCFR/c-Kit), **(H)** Common β chain (βc), **(I)** Hepatocyte growth factor receptor (HGFR/c-Met), **(J)** Sirtuin-1, **(K)** Granzyme A, **(L)** Myocyte-specific enhancer factor 2C (MEF2C), **(M)** Arginase-1, **(N)** DNA (cytosine-5)-methyltransferase 3A (DNMT3A), **(O)** Desmoglein-3, **(P)** Autotaxin (ENPP2), **(Q)** Hepatocyte growth factor **(R)**, Galectin-9, **(S)** Fas ligand (FASL), and **(T)** Pancreatic prohormone (PP). Expression levels are measured as protein concentrations (pg/mL) from the multiplex ELISA training dataset. Results are displayed as violin plots with dotted lines indicating the median and upper and lower quartiles. Data were analyzed by 1-way ANOVA (significance set to p<0.05) followed by Tukey's multiple comparison text (n ≥ 3 for all groups).

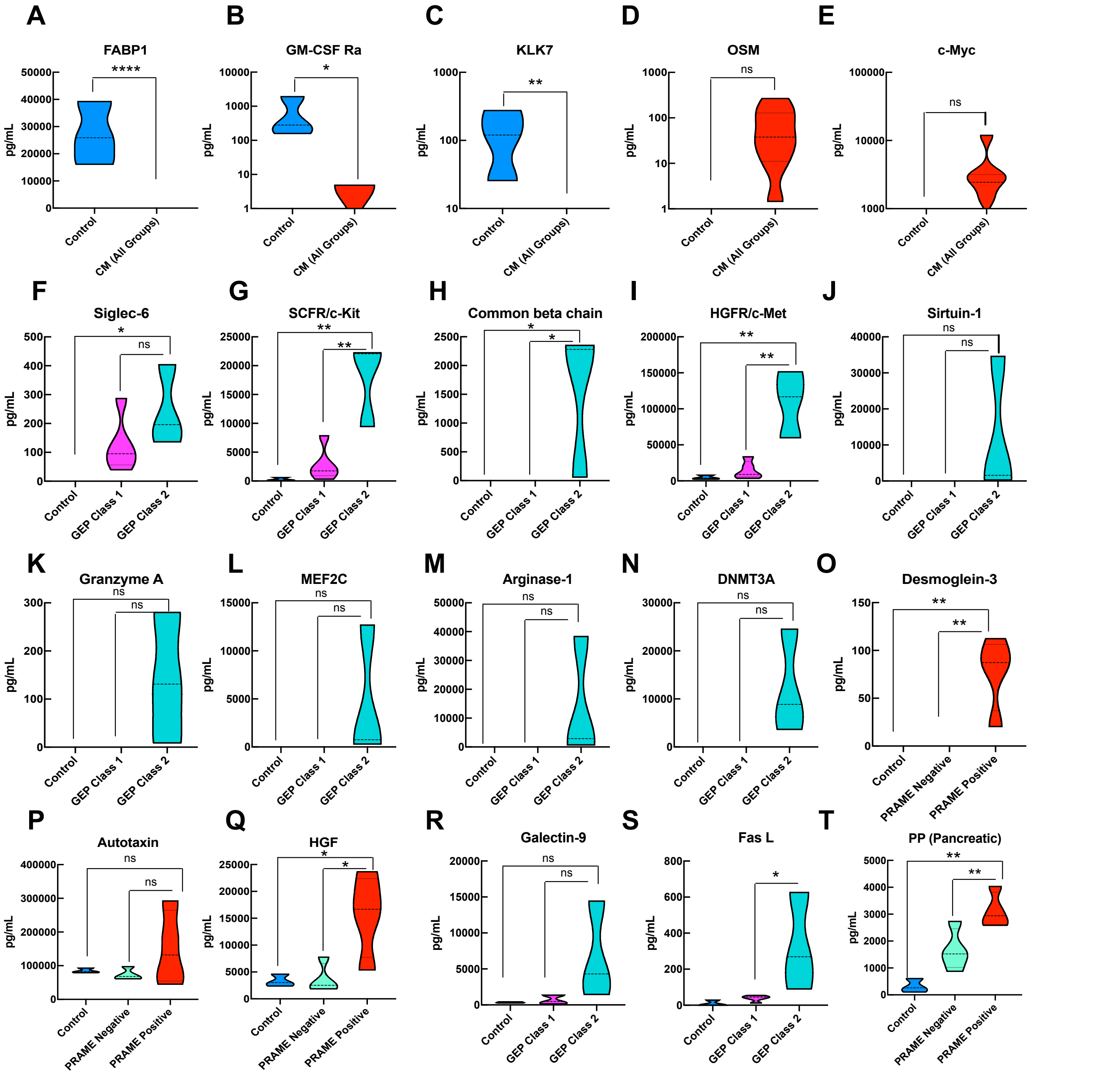

Fig. S2. Vitreous biomarker levels in verification dataset arranged by GEP Class: Protein expression levels from the verification study arranged by GEP Class. Expression levels are measured as protein concentrations (pg/mL) from the multiplex ELISA training dataset. Results are displayed as violin plots with dotted lines indicating the median and upper and lower quartiles. Data were analyzed by 1-way ANOVA (significance set to p<0.05) followed by Tukey's multiple comparison text (n ≥ 3 for all groups). Data is grouped by **(A)** predicted negative controls, **(B)** statistically significant markers, **(C)** trending markers (that did not display statistical significance), and **(D)** markers that did not display measurable trends.

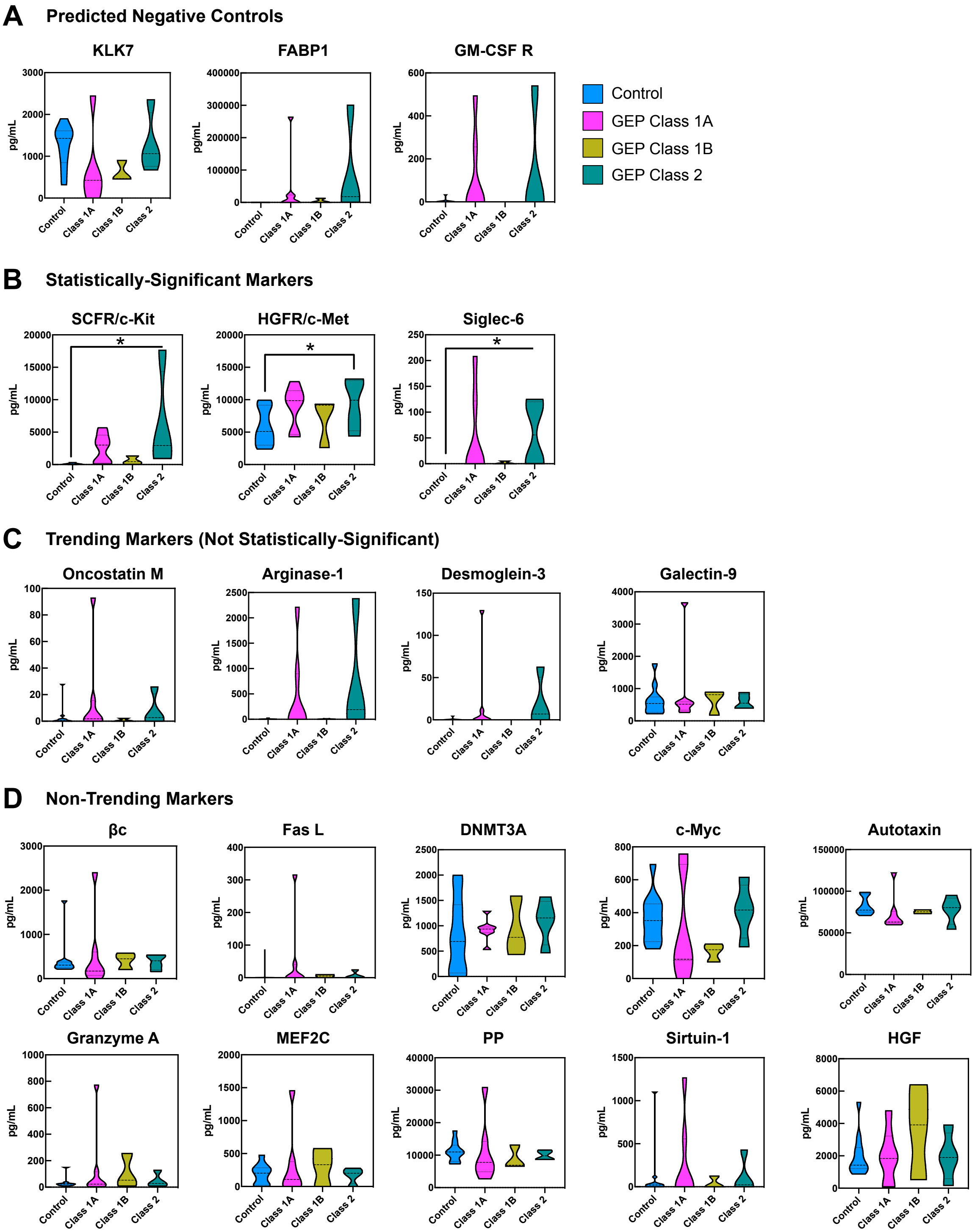

Fig. S3. Vitreous biomarker levels in verification dataset arranged by PRAME Status: Protein expression levels from the verification study arranged by PRAME status. Expression levels are measured as protein concentrations (pg/mL) from the multiplex ELISA training dataset. Results are displayed as violin plots with dotted lines indicating the median and upper and lower quartiles. Data were analyzed by 1-way ANOVA (significance set to p<0.05) followed by Tukey's multiple comparison text (n ≥ 3 for all groups). Data is grouped by **(A)** predicted negative controls, **(B)** statistically significant markers, **(C)** trending markers (that did not display statistical significance), and **(D)** markers that did not display measurable trends.

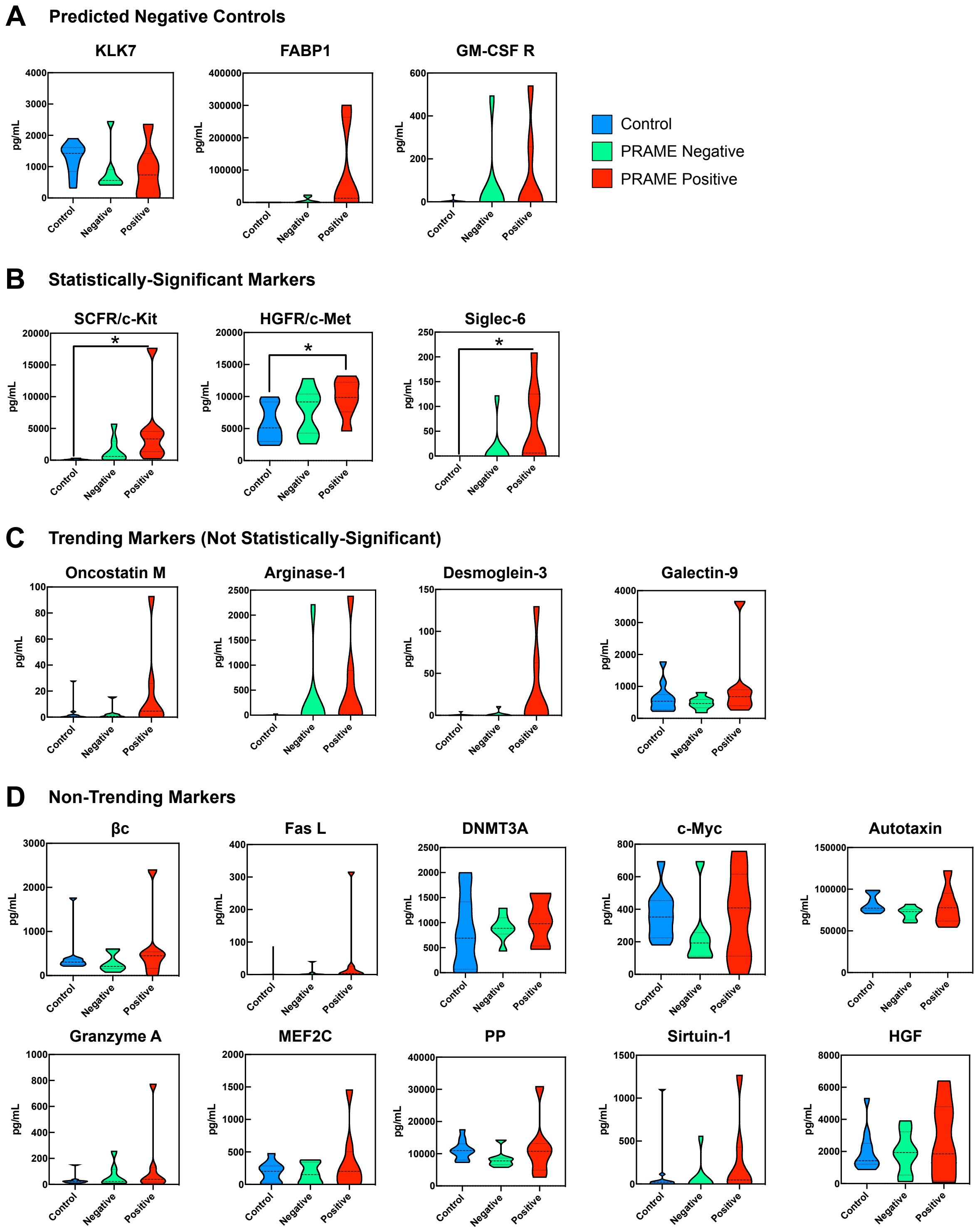

Fig. S4. Vitreous biomarker levels in verification dataset arranged by AJCC Stage: Protein expression levels from the verification study arranged by AJCC stage. Expression levels are measured as protein concentrations (pg/mL) from the multiplex ELISA training dataset. Results are displayed as violin plots with dotted lines indicating the median and upper and lower quartiles. Data were analyzed by 1-way ANOVA (significance set to p<0.05) followed by Tukey's multiple comparison text (n ≥ 3 for all groups). Data is grouped by **(A)** predicted negative controls, **(B)** statistically significant markers, **(C)** trending markers (that did not display statistical significance), and **(D)** markers that did not display measurable trends.

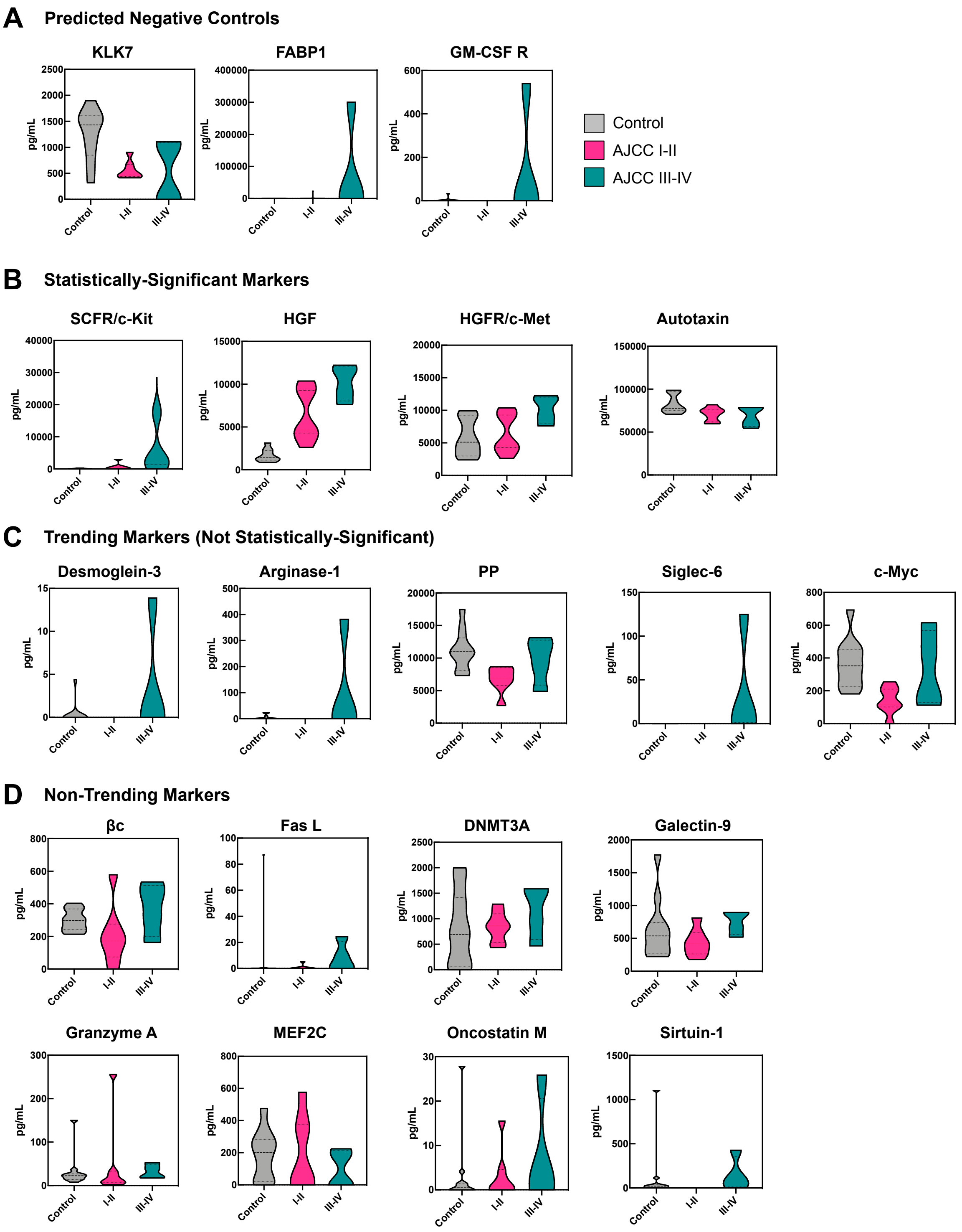

Supplement: Supplementary file 1 — Additional file 1. [file 12943_2021_1336_MOESM1_ESM.pdf]
